# Supplementary material for: Association between nurses’ knowledge and attitudes and caring behaviors toward people with opioid use disorder
Source: PLoS One. 2026 Mar 12;21(3):e0325874. doi: 10.1371/journal.pone.0325874 (PMC12981468; doi:10.1371/journal.pone.0325874)
Supplement: Attachment — Submitted filename: Comments 2.pdf [file pone.0325874.s002.pdf]

The article has been improved across several sections, starting from the title. The topic is relevant and novel. However, its main weakness lies in the methodology section, which leads to the generation of results lacking statistical rigor. The following comments are provided by section:

1. **Abstract:**
   This section can be reduced, as it exceeds 300 words.

2. **Introduction:**
   Improvements in the text are evident after the recent revisions. The section now reads more fluently and has a solid conceptual framework.

3. **Method:**

   - **Study Design:**
     It should be explicitly stated that this is a *quantitative* study.

   - **Study Measures:**
     Caring Behaviors: Instead of stating that Cronbach's alpha ranges "between 0.89 and 0.89," it is recommended to indicate that its Cronbach's alpha coefficient, reported across different studies, is 0.89.
     The variable measured by the CBI-6 is *discrete*. A discrete variable can only take integer, countable values — that is, it results from *counting*, not measuring.
     There are no intermediate values between two consecutive integers.
     Such variables are finite and integer-based; therefore, many parametric tests (such as Student's t-test or ANOVA) are not always appropriate, especially when the number of possible values is small or when the distribution is highly skewed.

For a Student's t-test to be valid, the following assumptions must be met:

1. The dependent variable is continuous or quasi-continuous (interval or ratio scale).
2. The distribution is approximately normal within each group.
3. Homogeneity of variances across groups.

If the discrete variable does not meet the assumptions of normality or continuity, nonparametric tests should be used instead. These tests do not require normality and are more robust for ordinal or discrete data.

Knowledge and Attitudes (DDPPQ):
This is also a discrete variable, so the same comment applies.

- **Covariates:**
  The decision to categorize continuous variables such as age and years of experience (e.g., 1–5 years, 6–10 years) for bivariate analyses may result in a loss of statistical power and information. If linear regression is used, it is

preferable to retain the continuous form of the variable—provided that the assumptions are met.

- **Data Analysis:**
  Unfortunately, the variables were treated as continuous, which is not appropriate since the CBI-6 and DDPPQ scores result from the summation of discrete Likert-type items. Furthermore, a normal distribution of data was assumed, which led to the use of parametric tests such as ANOVA and t-tests. It is important to note that nonparametric regression models and generalized linear models exist for discrete variables. Moreover, since both CBI and DDPPQ are evaluated through Likert-type scales, it would have been ideal to compute **polychoric correlations** between the variables.

4. **Results:**
   The tables show highly unbalanced group sizes for key variables. For instance: female = 114, male = 6, other = 1. This large disparity makes group comparisons statistically meaningless. The *p*-values in such cases have no interpretive validity.
   It is recommended that group comparisons be limited to **descriptive statistics** only. This issue affects most variables and leads to conclusions lacking statistical weight—not only due to unequal group sizes but also because the parametric tests used do not meet the required assumptions.
   As previously noted, it is suggested that the authors retain only **Research Question No. 2** and compute **correlations** applicable to the study sample. This approach would be more consistent with a **descriptive study design**.

5. **Discussion and Conclusions:**
   These sections should be adjusted to align with descriptive results, including correlation analyses.
