# Supplementary material for: Association between nurses’ knowledge and attitudes and caring behaviors toward people with opioid use disorder
Source: PLoS One. 2026 Mar 12;21(3):e0325874. doi: 10.1371/journal.pone.0325874 (PMC12981468; doi:10.1371/journal.pone.0325874)
Supplement: Attachment — Submitted filename: Authors.pdf [file pone.0325874.s004.pdf]

Dear Authors,

Although the manuscript has addressed some of the previously identified issues, the quantitative analysis remains very weak. Of particular concern are the assumptions underlying the data analysis plan.

The response variables measured by both instruments do not correspond to continuous variables. These data are obtained through Likert-type scales and, even when multiple items of this kind are summed, the resulting score does not constitute a numerical variable in the strict sense, nor a discrete variable in formal statistical terms, and certainly not a continuous one.

With respect to the ANOVA analysis, this term refers to a family of experimental designs that require the random assignment of study units to factors such as race, age group, nursing role, among others. None of these conditions are met in the present study. Causal inference would only be feasible if the formation of groups were truly random. Furthermore, it would be necessary to evaluate potential interactions among factors, ensure adequate balance across groups (which is also not the case in this manuscript), and verify that the data originate from an appropriate multivariate probability model—or a bivariate one if only two variables are analyzed. It is essential to determine whether the data genuinely conform to a multivariate model. Kim and Timm (2006) propose SAS routines to evaluate multivariate normality when the data are truly continuous; however, this assumption is not met here.

Likewise, it is not methodologically appropriate to apply Pearson correlation to data that are not continuous. Under these conditions, assessing normality is also meaningless, given that the variables are categorical and measured on an ordinal scale. For Likert-type variables with at least five categories, the appropriate technique for estimating associations is the polychoric correlation. Nevertheless, reliable estimation of polychoric correlations requires large sample sizes—typically exceeding 1,000 observations, based on simulation studies—which differs substantially from the 125 units reported in this study, a clearly insufficient number for any robust correlational analysis.

The widely circulated idea that "30 cases are sufficient" is a myth applicable only—if at all—and very narrowly to univariate variables. The required sample size depends on the variability of the variables and, in the case of correlations, on the magnitude of the population correlation. When the absolute value of the correlation between two variables is close to one, very few observations are needed; however, when correlations are close to zero, required sample sizes clearly exceed 1,000 data points. For multivariate studies, there are no explicit references supporting the use of multiple polychoric correlations with small sample sizes.

For all these reasons, and even though the limitations acknowledged by the authors are noted, the most appropriate course of action would be to present this work as a descriptive, exploratory study. It may serve as an initial step toward addressing this knowledge gap through future investigations with larger and randomly selected samples.

Finally, it is important to emphasize that nursing is a science and, as such, must adhere strictly to the methodological standards that guide the basic sciences. The quality of scientific evidence depends on the rigor and robustness of the procedures used. The fact that some authors in nursing research have employed questionable analytical strategies does not legitimize their use. It is the responsibility of the disciplinary scientific community to safeguard the quality and rigor of published research, thereby contributing to the strengthening and advancement of the field.

Lastly, I encourage you to review the following authors, whose work may help clarify the appropriate statistical approach for this study:

- Kiwanuka F, Kopra J, Sak-Dankosky N, Nanyonga RC, Kvist T. *Polychoric Correlation with Ordinal Data in Nursing Research*. Nursing Research. 2022;71(6):469–476.
- Kim, K., & Timm, N. (2006). *Univariate and Multivariate General Linear Models: Theory and Applications with SAS.* CRC Press.
