# Supplementary material for: Association between nurses’ knowledge and attitudes and caring behaviors toward people with opioid use disorder
Source: PLoS One. 2026 Mar 12;21(3):e0325874. doi: 10.1371/journal.pone.0325874 (PMC12981468; doi:10.1371/journal.pone.0325874)
Supplement: Attachment — Submitted filename: Letter to Editor.pdf [file pone.0325874.s005.pdf]

Dear Editor,

I have carefully reviewed the revised version of the manuscript. However, the methodological rigor, particularly the data analysis plan, remains insufficient, as it continues to rely on incorrect assumptions that lead to results lacking scientific robustness. It is essential to emphasize that a core component of any quantitative study is a data analysis plan that adheres to the procedures established within statistical science for the appropriate treatment and analysis of data.

In this case, the authors persist in applying analytical procedures that are not aligned with the nature of the data collected, resulting in conclusions that lack adequate theoretical support.

For these reasons and considering both the prestige of *PLOS ONE* and the responsibility to safeguard the quality of scientific publications in the field of nursing, my recommendation is to reject the manuscript in its current form.

I sincerely appreciate the trust that *PLOS ONE* has placed in me as a reviewer. I undertake this role with responsibility and commitment, recognizing that contributing to methodological rigor is fundamental to strengthening scientific knowledge within nursing discipline.

I hope to continue collaborating in the future to ensure that the journal's publications remain a significant contribution to scientific advancement.

Sincerely,

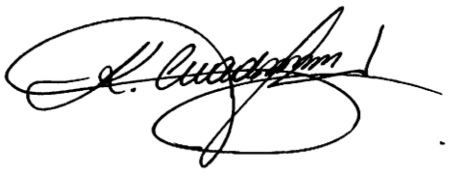

Katya Cuadros C. PhD
