# Supplementary material for: Association between nurses’ knowledge and attitudes and caring behaviors toward people with opioid use disorder
Source: PLoS One. 2026 Mar 12;21(3):e0325874. doi: 10.1371/journal.pone.0325874 (PMC12981468; doi:10.1371/journal.pone.0325874)
Supplement: Attachment — Submitted filename: PONE_25_26604_Edits Letter Response.docx [file pone.0325874.s001.docx]

PONE-D-25-26604

Influence of Nurse’s Knowledge and Attitudes on Caring Behaviors for People with Opioid Use Disorder

Ph.D. Inyene Edem Essien-Aleksi

Dear Dr. Essien-Aleksi,

We've checked your submission and before we can proceed, we need you to address the following issues:

1. In the Ethics Methods section of your manuscript, please state whether informed consent was written or verbal. If consent was verbal, please explain: a) why written consent was not obtained, b) how you documented participant consent, and c) whether the ethics committee/IRB approved this consent procedure.

**Answer: Thank you for your swift feedback. I have updated the method section to include specifics about the written informed consent process for the study.**

2. Please complete all items on the Human Participants Research Checklist that are relevant for your submission, by following this link:  <http://journals.plos.org/plosone/s/file?id=dc11/PLOSOne_Human_Subjects_Research_Checklist.docx> (Contact us at [plosone@plos.org](mailto:plosone@plos.org) if you cannot access the document.) There may be overlap between the checklist items and other queries listed below; please address any duplicated queries both in your response email and on the checklist itself. Upload the completed Human Participants Research Checklist as file type “Other” when you re-submit your manuscript. This document is for internal journal use only and will not be published if your article is accepted. The requested information will help us to assess whether your submission complies with PLOS ONE’s policies and adheres to applicable reporting standards. Note that your manuscript may be rejected if you provide incomplete or inadequate responses to the checklist questions and that changing the

‘Section/Category’ of your article does not affect this requirement.

**Answer: I have completed the checklist and included the following documents: the original IRB approval forms from Tufts Medicine and Merrimack College; the resubmission/modification form submitted to Tufts Medicine to extend the study recruitment period to December 2024; and the approved study protocol (Form 7). Thank you.**

We've returned your manuscript to your account. Please resolve these issues and resubmit your manuscript within 21 days. If you need more time, please email the journal office at [plosone@plos.org.](mailto:plosone@plos.org.) We are happy to grant extensions of up to one month past this due date. If we do not hear from you within 21 days, we will withdraw your manuscript.

Please log on to PLOS Editorial Manager at <https://www.editorialmanager.com/pone/> to access your manuscript. You will find your manuscript in the 'Submissions Sent Back to Author' link under the New Submissions menu. Be sure to remove your previous manuscript file if you are uploading a new file in response to these requests. After you've made the changes requested above, please be sure to view and approve the revised PDF after rebuilding the PDF to complete the resubmission process.

We are requesting these changes to comply with the PLOS ONE submission guidelines (https://journals.plos.org/plosone/s/submission-guidelines). Please note that we won't send your manuscript for review until you have resolved the above requests.

Thank you for submitting your work to PLOS ONE and supporting our mission of Open Science.

Kind regards,

Irene Nathalie Fernandez Tolentino

PLOS ONE

__________________________________________________

In compliance with data protection regulations, you may request that we remove your personal registration details at any time.  (Use the following URL: https://www.editorialmanager.com/pone/login.asp?a=r). Please contact the publication office if you have any questions.
