# Supplementary material for: Association between nurses’ knowledge and attitudes and caring behaviors toward people with opioid use disorder
Source: PLoS One. 2026 Mar 12;21(3):e0325874. doi: 10.1371/journal.pone.0325874 (PMC12981468; doi:10.1371/journal.pone.0325874)
Supplement: Attachment — Submitted filename: 3. Response to Reviewers.docx [file pone.0325874.s003.docx]

**Manuscript Number:** PONE-D-25-26604

**Title:** Association Between Nurses’ Knowledge and Attitudes and Caring Behaviors Toward People with Opioid Use Disorder

Dear Editor and Reviewers,

We sincerely appreciate your valuable comments. We have reviewed the suggestions from reviewers 1-5 and updated the manuscript accordingly. Please see our responses below in bold and in the manuscript for more details.

Sincerely,

Inyene Essien-Aleksi, PhD.

**Response to the Editor**

1. Please ensure that your manuscript meets PLOS ONE's style requirements, including those for file naming. The PLOS ONE style templates can be found at
   https://journals.plos.org/plosone/s/file?id=wjVg/PLOSOne_formatting_sample_main_body.pdfand <https://journals.plos.org/plosone/s/file?id=ba62/PLOSOne_formatting_sample_title_authors_affiliations.pdf>]

**We have formatted the manuscript to ensure it meets PLOS ONE’s style requirements.**

2. Please note that funding information should not appear in any section or other areas of your manuscript. We will only publish funding information present in the Funding Statement section of the online submission form. Please remove any funding-related text from the manuscript.

**We have revised the manuscript to ensure that no funding information is included anywhere. We received** **no funding from federal agencies or private organizations for this project.**

We note that the grant information you provided in the ‘Funding Information’ and ‘Financial Disclosure’ sections do not match. When you resubmit, please ensure that you provide the correct grant numbers for the awards you received for your study in the ‘Funding Information’ section.

**We have updated the Funding Statement and Financial Disclosure section to state that we received no funding from federal agencies or private organizations for this project.**

3. When completing the data availability statement of the submission form, you indicated that you will make your data available on acceptance. We strongly recommend all authors decide on a data sharing plan before acceptance, as the process can be lengthy and hold up publication timelines. Please note that, though access restrictions are acceptable now, your entire data will need to be made freely accessible if your manuscript is accepted for publication. This policy applies to all data except where public deposition would breach compliance with the protocol approved by your research ethics board. If you are unable to adhere to our open data policy, please kindly revise your statement to explain your reasoning and we will seek the editor's input on an exemption. Please be assured that, once you have provided your new statement, the assessment of your exemption will not hold up the peer review process.

**Thank you for your comment.** **All relevant study data (de-identified) are now freely accessible. The data have been uploaded into the openICPSR data repository, with the link provided below.  [https://www.openicpsr.org/openicpsr/project/238465/version/V1/view](https://www.openicpsr.org/openicpsr/project/238465/version/V1/view)**

Please include your full ethics statement in the ‘Methods’ section of your manuscript file. In your statement, please include the full name of the IRB or ethics committee who approved or waived your study, as well as whether or not you obtained informed written or verbal consent. If consent was waived for your study, please include this information in your statement as well.

**The method section has been revised to include the following information: “**Permission to conduct this study was obtained from the Institutional Review Board (IRB) of Merrimack College (IRB -FY24-25-2) and Tufts Medicine (IRB #00004941). To collect data, the research team obtained electronic written informed consent from the participant prior to recruitment and data collection	**.”**

We note you have included a table to which you do not refer in the text of your manuscript. Please ensure that you refer to Table 4 in your text; if accepted, production will need this reference to link the reader to the Table.

**Thank you. We have revised the manuscript to include a reference to Table 4 in the Results section.**

4. If the reviewer comments include a recommendation to cite specific previously published works, please review and evaluate these publications to determine whether they are relevant and should be cited. There is no requirement to cite these works unless the editor has indicated otherwise.

**Thank you for the clarification. No reviewer recommended citing specific previously published works. However, we reviewed the manuscript's in-text citations and ensured the most relevant references are included to strengthen it.**

**Response to the Editor and Reviewer(s)**

1. **Reviewer #1:** First of all, the topic is very interesting and distinctive and deserves recognition. However, scientific writing contains many mistakes. There are a lot of repetitions in the abstract part, and it is very long. There are many incorrect references in the introduction, and the references are not numbered correctly and do not start with number one. The method section lacks clarity. To make the results more clear, the tables need to be accompanied by commentary. Furthermore, there is no explanation as to the data availability.

**Thank you, Reviewer #1, for your thoughtful and constructive feedback. We have made the following revisions to the manuscript: 1) Abstract: Significantly revised to eliminate repetition and shortened to meet PLOS ONE publishing guidelines. 2) Introduction: Corrected citation errors and ensured the references are numbered sequentially, starting at 1. 3) Methods: Revised for better clarity. 4) Results: Updated tables with additional commentary to enhance interpretation and clarity. 5) Data Availability: We have included a link to the** **openICPSR data repository in both the Supporting Information (within the manuscript) and the online submission form.**

2. Reviewer #2: The manuscript is clearly written and facilitates reader comprehension. Nevertheless, the authors are encouraged to use and refer to a standardized checklist to further improve the quality and transparency of the methodology section.

**Thank you, Reviewer #2, for your feedback. We have made revisions to improve the quality of the method section following the criteria outlined in PLOS ONE’s standardized checklist.**

3. Reviewer #3: This manuscript addresses a highly relevant clinical and public health topic: the impact of nurses’ knowledge and attitudes on their caring behaviors toward patients with opioid use disorder (OUD). The study employs validated instruments (DDPPQ and CBI-6) and appropriate statistical methods. However, some methodological and reporting weaknesses must be addressed to strengthen the study’s rigor, reproducibility, and generalizability.

   Participants and Sample
   Observation: Inclusion/exclusion criteria are described, but no justification or power calculation for sample size is provided. Recommendation: Include a rationale or power analysis for the sample size, or acknowledge this as a limitation.

**Thank you, Reviewer #3, for all your comments and constructive feedback. We have revised the data analysis paragraph to specify that power analysis was performed using G*Power and that** **a total of 84 participants were needed to detect a medium effect size (d = 0.50) with 80% power at a 0.05 significance level.**

Bias Observation: There is no discussion of potential selection bias, self-selection bias, or social desirability bias due to self-reporting and voluntary participation. Recommendation: Address these biases in the limitations section and suggest mitigation strategies for future research (e.g., random sampling, cross-validation, stronger anonymity).

**We agree with Reviewer #3 concerning the potential for biases. In the limitations section, we have now emphasized the potential for response bias, self-selection bias, and social desirability bias, which are inherent to the self-reporting nature of the instruments used in the study and the voluntary participation. We have suggested mitigation strategies for future studies, including random sampling, as well as cross-validation in a multi-site research framework to protect participants’ confidentiality/anonymity.**

Measurement and Analysis
Observation: The use of validated instruments and appropriate statistical techniques is commendable. However, as all measures are self-reported, there is a risk of bias. Recommendation: Discuss the potential for response bias (e.g., overreporting of caring behaviors) due to the self-reported nature of both primary instruments.

**We agree with Reviewer #3 about the risk of response bias. We highlighted this risk due to the self-reporting measures and described strategies to mitigate this limitation in the discussion section.**

Causal Inference
Observation: Some wording in the discussion may suggest causal relationships. Recommendation: Reinforce that, given the cross-sectional design, only associations—not causal relationships—can be inferred.

**We agree with Reviewer #3 for your comments regarding causal inference. We have thoroughly revised the discussion, limitations, and conclusion sections to include more specific language, indicating that our findings reflect only associations and not causal relationships. We have also changed the study title from “ Influence” to “ Association Between Nurses’ Knowledge and Attitudes and Caring Behaviors Toward People With Opioid Use Disorder”.**

**Reviewer #4: Abstract**
The abstract attempts to summarize a dense and multifaceted study but becomes cluttered with too much background. The research objective gets somewhat buried. The phrasing “Additionally, we further examined differences...” is redundant. Reporting results with specific statistics (β = -0.11, p < 0.0001) in the abstract is good, but the implications of these findings are not adequately synthesized. There’s minimal interpretation beyond stating the association. The phrase “Nurses’ knowledge and attitudes play a crucial role...” is repeated in the abstract and again in the conclusion with near-identical wording, suggesting a lack of synthesis.

**Thank you, Reviewer #4 for your construction feedback. We have significantly revised the abstract to remove repetition and ensure the findings are well synthesized and more concise.**

**Introduction**
The introduction excessively relies on citations (e.g., multiple references in one sentence) without critically integrating them. Some references seem included for volume rather than relevance.
Although the issue is important, the introduction lacks a clear conceptual model or hypothesis linking knowledge, attitudes, and caring behavior.
The gap in the literature is stated but not well substantiated. The claim that this is the “first study” to link these constructs lacks sufficient justification or contrast with existing studies.
Terms like “caring behavior” and “attitudes” are introduced without conceptual definitions, relying instead on the instruments (CBI-6, DDPPQ) to carry the burden of meaning.

**We appreciate your thoughtful feedback. We have revised the introduction section to strengthen the integration of sources, refine the statement about the gap, and provide definitions of caring behaviors and attitudes based on previous studies. Since a formal hypothesis is not mandated for a descriptive cross-sectional study, our research was exploratory in nature, with specific research questions aimed at investigating demographic and work-related factors that could show differences in nursing knowledge, attitudes, and caring behaviors, as well as how these factors may influence the relationship between these two variables. Future longitudinal and interventional studies with diverse and large samples are needed to explicate whether and how these factors influence caring behaviors.**

**Methods**
The use of convenience and purposive sampling is a significant limitation, but this is not acknowledged until the discussion. This undermines generalizability and introduces bias.
The exclusion of nurse managers and educators is reasonable but not well-justified. Given their role in shaping attitudes and policy, their exclusion may miss important systemic insights.
The explanation of the DDPPQ scoring is confusing. The creation of stratified ranges (e.g., “very low,” “low,” etc.) seems arbitrary without validation or citation.
The methods mention the use of REDCap and a raffle incentive, but there's no reflection on how these may introduce self-selection or response bias.
No justification for the sample size is provided, which weakens confidence in the representativeness and power of statistical analyses.

**We have thoroughly revised the method section. Since there are no specific cutoff points for DDPPQ and CBI-6, the research team’s stratification of the instruments was exploratory in nature. Both the independent and dependent measures were used as continuous variables [total scores] when testing the association between the variables. We revised the manuscript to remove mentions of stratification. Instead, we only kept references to the distribution of the measure [range] and internal consistency testing for the studied sample. We also acknowledged the implications of the raffle incentive process via REDcap. Although participation was voluntary, we recognize that motivated participants interested in addiction medicine or research may have been more likely to respond. Incentives may have also increased participation, motivating some participants to complete the survey. To minimize self-selection and response biases, we randomly select raffle winners through an electronic system. Additionally, we ensured that the survey responses were kept separate from the incentive distribution protocol.**

**Results**
The section is filled with statistical comparisons but lacks interpretation. Reporting p-values and CI is appropriate, but there’s minimal explanation of what these mean practically.
The categorization of DDPPQ and CBI-6 scores into subjective ranges (e.g., high, moderate, low) is not grounded in prior literature, reducing replicability.
The volume and complexity of the tables may overwhelm the reader. Many differences are noted as statistically insignificant, suggesting a risk of fishing for significance.
The emphasis on statistical significance ignores potential Type I errors due to multiple comparisons without correction.

**Thank you, reviewer 4. In response to your comments regarding the method and results sections, we have revised these sections to strongly highlight all the potential limitations related to convenience and purposive sampling. Additionally, we updated the methods section to include reasons for excluding non-bedside nurses. Since DDPPQ and CBI-6 tools do not have established cut-off points, we conducted a descriptive exploration of the instruments' distribution by creating categorical subunits for each scale. However, we kept the dependent and independent variables as continuous measures. As a result, we have removed the text related to the stratification of both measures from the manuscript. Regarding the tables, several research studies have identified multiple demographic and workplace factors associated with substance use disorder. This study aimed to examine whether some of these factors would show differences in the specific population of patients with opioid use disorder. Only a few showed some differences.**

Discussion
The discussion makes strong claims about causality and implications despite the cross-sectional design. Phrases like “nurses’ knowledge and attitudes were the strongest predictors” suggest a causal relationship unsupported by the study design.
Many points are reiterated from earlier sections (e.g., “nurses in community hospitals had less favorable attitudes”), which inflates word count without adding insight.
Although some literature is cited, there’s a lack of critical integration. The findings are not well-situated within broader theoretical or empirical contexts.
Statements about how institutional culture or training might affect outcomes are speculative and unsupported by data from the study.
The limitations section is undercut by generic statements. The gender imbalance and response bias deserve deeper examination.

**Thank you, Reviewer 4, for your insightful feedback. We have carefully revised the discussion section to remove causal language. We also made additional changes to better integrate the literature and eliminate redundancy. Furthermore, we strengthened the limitations section and addressed the gender imbalance in our sample compared to the national nursing workforce.**

Conclusion and Implications
The conclusion pushes the policy relevance too hard without sufficient data to support its sweeping claims (e.g., EMR-integrated interventions).
Recommendations for educational interventions are vague. What kind of intervention? Delivered how? By whom?
The conclusion asserts that RN licensure status “contributes” to caring behavior, yet the earlier analysis shows only a weak association. The term “contributes” overstates the evidence.
Ethical and Data Transparency Statements
The ethics section is sufficiently detailed but fails to address potential coercion or social desirability bias given that recruitment was conducted internally and involved monetary incentives.
While the link to OpenICPSR is appreciated, the manuscript does not indicate whether de-identified datasets were reviewed for completeness or whether codebooks are available.

References
Several references are dissertations or lesser-known journals, which may weaken the scholarly weight. Peer-reviewed, high-impact studies should be prioritized.
Some studies are cited multiple times in different sections with near-identical phrasing, indicating potential padding.

**Thank you for your insights on the conclusion section. We have thoroughly revised this section to incorporate implications and integrate relevant literature supporting our findings. We removed causal language and added two strategies — empathy-based and humanistic nursing education/approaches — which have been tested and validated to improve nursing knowledge and attitudes in SUD/OUD intervention studies. We updated the method and limitation section to address concerns related to incentives, recruitment strategies, and potential biases. All relevant study data (de-identified) are now freely accessible. The data have been uploaded to the openICPSR data repository, with the link provided in the supporting document of the manuscript and the online submission form. The codebook will be made available upon request. Unfortunately, since few OUD-specific studies have been conducted to examine the effects of knowledge, attitudes, and caring among hospital nurses, our reference relied on diverse journals, including dissertations, international journals, and lesser-known publications. This is why this study is essential, as exploring this understudied topic is needed in the nursing field.**

**Reviewer #5: 1. Title vs. Study Design**
There is a fundamental inconsistency between the study title and the actual research design. The title implies a causal relationship (“influence”), whereas the study employs a cross-sectional, observational design using correlational analyses. Causal inference is not warranted in such a design.
Recommendation: Consider revising the title to reflect the observational nature of the study. For example:
“Association Between Nurses’ Knowledge and Attitudes and Caring Behaviors Toward People With Opioid Use Disorder”, or
“Exploring the Relationship Between Nurses’ Knowledge and Attitudes and Their Caring Behaviors...”

**Thank you, Reviewer 5, for your insightful recommendation. We have revised the title to “Association Between Nurses’ Knowledge and Attitudes and Caring Behaviors Toward People with Opioid Use Disorder”.**

2. Clarity and Alignment of Objectives
The study's aim is not clearly and explicitly stated. The main objective appears to be to examine the relationship between nurses’ knowledge/attitudes and caring behaviors, while a secondary objective is to explore differences based on demographic and workplace factors. However, the latter is distorted by an erroneous analysis plan.

**Thanks for your recommendation. We have revised the introduction section to emphasize our research question and the method section to include the aim of your study. In the Results section, we have included a reference explaining why the study was conducted. We used an independent t-test to compare the means of the continuous DDPPQ and CBI-6 scores between the two groups, based on either demographic or workplace variables. For more than two groups, one-way ANOVA was employed, followed by pairwise comparisons.**

3. Study Design and Sampling
The study is described as cross-sectional, which is appropriate for examining associations. However, the wording in other parts of the manuscript (e.g., title and conclusions) suggests causal inference. This discrepancy should be addressed throughout to avoid overinterpretation of findings.
The use of convenience and purposive sampling introduces potential bias, especially regarding representativeness. While this is common in exploratory nursing studies, it limits generalizability and should be acknowledged.
Recommendation: Clarify whether a power analysis or sample size estimation was conducted a priori. If not, provide a rationale for the achieved sample size and its implications for statistical power.

**Thank you. We have updated the method section to include that power analysis was conducted using G*power. 84 Participants were needed to detect a medium effect size (d = 0.50) with 80% power at a 0.05 significance level. We have thoroughly revised the conclusion section to remove wording related to cause/effect relationship. The limitation section now addresses potential recall bias, attrition bias, social desirability bias, and selection bias. Mitigation strategies to reduce these biases have been discussed.**

3. Measurement of Key Constructs
a. Caring Behaviors (Dependent Variable)
The use of the Caring Behavior Inventory-6 (CBI-6) is appropriate and supported by internal consistency data. However, the categorization of CBI-6 scores into “low, moderate, and high” levels appears arbitrary, as the instrument lacks validated cut-off points. Correct the range of Cronbach's alpha of the CBI-6
Recommendation: Justify the rationale for the chosen score ranges, or alternatively, consider treating the CBI-6 score as a continuous variable in line with existing literature.
b. Knowledge and Attitudes (Independent Variable)
The DDPPQ is used as a combined measure of nurses’ "knowledge and attitudes." While this usage is consistent with some prior studies, the DDPPQ is more accurately described as a measure of perceived therapeutic competence and attitudes, not direct knowledge.
The stratification into “very low” to “high” levels based on Likert scale anchors is methodologically problematic, as DDPPQ has no validated thresholds and its interpretation is context sensitive.
Recommendation: Clearly distinguish between attitudes, role adequacy, and knowledge, as they are distinct theoretical constructs. If using the total DDPPQ score, provide a strong conceptual rationale and avoid over-interpreting results in terms of factual knowledge.

**Thank you. The DDPPQ and CBI-6 tools lack established cut-off points. We performed a descriptive analysis of the instruments' distribution by creating categorical subunits for each scale in our study sample. However, we maintained the dependent and independent variables as total scores and continuous variables in our analysis. For example, several studies have used total scores, subscale scores, or divided the items of the DDPPQ into knowledge and attitude dimensions [30, 31]. There has been no clear rationale for the varied measurement approaches, other than to assess and validate dimensions of the subscales. We chose to use total scores or combined measures of knowledge and attitudes, as this aligns with how the scale has been employed in previous studies.**

4. Recruitment and Ethical Procedures
Recruitment strategies and informed consent procedures are clearly described. The inclusion of a HIPAA-compliant recruitment platform and IRB approvals enhance the ethical credibility of the study.
Recommendation: It may be helpful to briefly comment on how the use of multiple recruitment methods may have influenced sample composition (e.g., risk of self-selection bias).

**Thank you. We have included that we used multiple recruitment methods (e.g., email, flyers, mobile app, meetings, and in-person) to minimize recruitment and selection biases and to reach a diverse group of participants. However, randomly selecting the target population would be much more effective in reducing selection biases.**

5. Data analysis plan. Although the manuscript reports p-values, these should be interpreted solely as descriptive indicators within the context of the study's design. The use of t-tests and one-way ANOVA implies assumptions of normality in the distribution of the response variable—assumptions that are not justified in this case. The variables analyzed are based on composite scores from Likert-type items, which do not warrant the application of parametric methods. Non-parametric approaches would be more appropriate for this type of data.
In addition, the study lacks an experimental or quasi-experimental design that would allow for valid group comparisons (e.g., by sex, ethnicity, or type of hospital). Since these group characteristics are not randomly assigned, the comparisons presented in the manuscript are not supported by the design and cannot be interpreted as causal.
Therefore, this manuscript should be considered as presenting preliminary descriptive findings. To support hypothesis testing and group comparisons, a follow-up study with a design that allows for such analyses would be necessary. For instance, an experimental design could include an intervention such as standardized training for healthcare personnel—an element not present in the current study.
Ensure the analytical strategy is fully transparent, including model types and rationale for covariate adjustment.
Clarify the interpretive boundaries of the design, particularly in relation to the causal language used elsewhere in the manuscript.

**Thank you. We have now clarified that the descriptive cross-sectional study design prevents any determination of cause and effect. We have highlighted all potential limitations and recommend a follow-up longitudinal or interventional study design that enables analysis of causality to be conducted. We included in the data analysis and covariates section that explored all the stated demographic and work-related factors, as they relate to the main measures, and covariates significantly associated with both the independent and dependent variables were adjusted in the regression analysis as confounding variables.**

6. Results
The final analytic sample represents approximately 56% completion (125/224), which raises concerns about response bias. No information is provided about differences between completers and non-completers, which could affect the generalizability of findings.
Main Association: Knowledge/Attitudes and Caring Behaviors: The study reports a significant negative association between DDPPQ scores and CBI-6 scores, in both unadjusted and adjusted models. The use of simple linear regression (Model 1) followed by a generalized linear model (Model 2) with covariates may be methodologically acceptable. However, the term “Generalized Linear Model” is too broad and lacks specificity (e.g., link function, distribution). It is also unclear whether the assumptions of linearity, normality, and homoscedasticity were tested. The reported β coefficients are statistically significant but small in magnitude (β = -0.11 to -0.12), which should temper interpretations of practical significance.
DDPPQ and CBI-6 were stratified into categories (e.g., low/moderate/high), despite the lack of validated cut-offs for either scale. This raises concerns about data-driven categorization and the risk of artificial thresholds, which may reduce statistical power and obscure true relationships.
The finding that RNs scored higher on CBI-6 than nurse educators is statistically significant but may reflect differences in direct patient care exposure rather than intrinsic caring capacity.
The conclusion that holding an RN license contributes to caring behaviors may be an overinterpretation, especially given the small group size and possible confounding by role responsibilities.
The results section presents statistically significant and potentially meaningful findings. However, several issues require attention: Clarity in model specification and assumptions, Justification for categorization of continuous measures, Cautious interpretation of subgroup findings and their implications, Avoidance of causal language in describing observational associations.

**We have revised the results section for clarity and eliminated the categorization of continuous measures, since both measures were analyzed as continuous variables in the regression analyses. We removed language implying causality and instead highlighted the reported relationship and association, while acknowledging potential limitations and the need for further research. Additionally, we have redefined the variable RN license to RN role. This is more representative of the distinctions between the categorical options measured in the variable (LPN, RN, nurse educator).**

7. Discussion
Add a paragraph analyzing possible reasons why experience and age did not show an association with attitudes and knowledge, contrasting with prior literature. For example, cultural differences, generational changes in training, or sample bias could be considered.
Elaborate more on the potential influence of professional role on caring behaviors, taking into account direct patient contact, workload, or level of ongoing professional development.

Include a comment on the gender representativeness of the sample relative to the national or regional nursing workforce demographics, which would help clarify the extent of this limitation.
Consolidate repetitive ideas to improve cohesion and avoid the reader perceiving redundancy.
Propose clear directions for future research, ideally focusing on how to overcome current limitations and deepen understanding of the factors shaping attitudes and behaviors related to OUD care.

**We have thoroughly revised the discussion, adding potential reasons why experience and age did not show an association. We revise and consolidate repeated ideas to improve cohesion and propose directions for future research that align with the study findings and limitations. Thank you for your insightful comments.**
