# Supplementary material for: Association between nurses’ knowledge and attitudes and caring behaviors toward people with opioid use disorder
Source: PLoS One. 2026 Mar 12;21(3):e0325874. doi: 10.1371/journal.pone.0325874 (PMC12981468; doi:10.1371/journal.pone.0325874)
Supplement: Attachment — Submitted filename: 1. Response to Reviewers (2).docx [file pone.0325874.s006.docx]

**Manuscript Number:** PONE-D-25-26604

**Title:** Association Between Nurses’ Knowledge and Attitudes and Caring Behaviors Toward People with Opioid Use Disorder

Dear Editor and Reviewers,

We sincerely appreciate your additional comments. We have reviewed Reviewer Five's suggestions and made the necessary updates to the manuscript accordingly. Please see our responses below in bold and in the manuscript for more details.

Sincerely,

Inyene Essien-Aleksi, PhD.

**Review Comments to the Author**

Please use the space provided to explain your answers to the questions above. You may also include additional comments for the author, including concerns about dual publication, research ethics, or publication ethics. (Please upload your review as an attachment if it exceeds 20,000 characters)

**Reviewer #1**: (No Response)

**Thank you for your feedback.**

**Reviewer #3:** A power analysis was conducted using G*Power to justify the sample size. The discussion was expanded to address potential response bias, and causal language was carefully avoided throughout the manuscript.

**Thank you for your comment.**

**Reviewer #5:** The article has been improved across several sections, starting from the title. The topic is relevant and novel. However, its main weakness lies in the methodology section, which leads to the generation of results lacking statistical rigor. The following comments are provided by section:

1. Abstract:
This section can be reduced, as it exceeds 300 words.

**We have revised the abstract to limit its length to 300 words. Thank you.**

**2. Introduction:**
Improvements in the text are evident after the recent revisions. The section now reads more fluently and has a solid conceptual framework.

**Thank you for your valuable feedback, which greatly enhanced the introduction section of this paper.**

**3. Method:**
o Study Design: It should be explicitly stated that this is a quantitative study.

**Thank you for your comment. In the first sentence of the methods section, we now specify that this is a “quantitative cross-sectional study…”**

o Study Measures: Caring Behaviors: Instead of stating that Cronbach’s alpha ranges “between 0.89 and 0.89,” it is recommended to indicate that its Cronbach’s alpha coefficient, reported across different studies, is 0.89.

**Thanks for your comment. I have updated the sentence to say that “the Cronbach’s α reported across various studies for the CBI-6 was 0.89.”**

The variable measured by the CBI-6 is discrete. A discrete variable can only take integer, countable values — that is, it results from counting, not measuring. There are no intermediate values between two consecutive integers. Such variables are finite and integer-based; therefore, many parametric tests (such as Student’s t-test or ANOVA) are not always appropriate, especially when the number of possible values is small or when the distribution is highly skewed.
For a Student’s t-test to be valid, the following assumptions must be met:
1. The dependent variable is continuous or quasi-continuous (interval or ratio scale).
2. The distribution is approximately normal within each group.
3. Homogeneity of variances across groups.
If the discrete variable does not meet the assumptions of normality or continuity, nonparametric tests should be used instead. These tests do not require normality and are more robust for ordinal or discrete data. Knowledge and Attitudes (DDPPQ):
This is also a discrete variable, so the same comment applies.

**Thank you for your insightful feedback. The DDPPQ and CBI-6 are both Likert-type scales, which are ordinal at the single-item level, but the composite scores were calculated as the sum of all items in the tools. In nursing research, we generally treat total scores from such scales as continuous (interval-level) data for statistical analysis. For instance, Watson et al. (2007) and Wu et al. (2006) analyzed DDPPQ and CBI-6 scores as continuous variables. Similarly, other studies have followed this approach when examining the psychometric properties and applications of the CBI, including Coulombe et al. (2002) and Edvardsson et al. (2015). These precedents support our decision to treat DDPPQ and CBI-6 scores as continuous rather than categorical or discrete variables.**

**References:**

- Watson, H., Maclaren, W., & Kerr, S. (2007). Staff attitudes towards working with drug users: Development of the Drug Problems Perceptions Questionnaire. Addiction, 102(2), 206–215.
- Wu, Y., Larrabee, J. H., & Putman, H. P. (2006). Caring Behaviors Inventory: A reduction of the 42-item instrument. Nursing Research, 55(1), 18–25.
- Coulombe, K. H., Yeakel, S., Maljanian, R., & Bohannon, R. W. (2002). Caring Behaviors Inventory: Analysis of responses by hospitalized surgical patients. Outcomes Management, 6(3), 138–141.
- Edvardsson, D., Mahoney, A. M., Hardy, J., McGillion, T., McLean, A., Pearce, F., et al. (2015). Psychometric performance of the English language six-item Caring Behaviours Inventory in an acute care context. Journal of Clinical Nursing, 24(17–18), 2538–2544.

Covariates:
The decision to categorize continuous variables such as age and years of experience (e.g., 1–5 years, 6–10 years) for bivariate analyses may result in a loss of statistical power and information. If linear regression is used, it is preferable to retain the continuous form of the variable—provided that the assumptions are met.

**Thank you for this insightful comment. It is true that categorizing continuous variables, such as age and years of experience, can reduce statistical power and information. In this study, we used a generalized linear regression model (multiple linear regression) and treated the independent and dependent variables as continuous, while controlling for relevant demographic and work-related factors. The reason we categorized certain covariates, such as age and years of experience, was to examine meaningful group differences and inform future interventions targeting specific groups. For example, whether nurses in certain age groups or with specific years of experience may require more attention or interventions. This approach is also supported by previous studies. For instance, Kravotil et al. (2023) showed that older nurses with more years of nursing experience (stratified into discrete variables) tended to have greater knowledge and more positive attitudes than their younger counterparts (although not consistently across all studies). Stratifying these work-related factors enabled us to balance statistical rigor with practical and empirical implications.**

**Reference:**

- Kratovil A, Schuler MS, Vottero BA, Aryal G. Nurses' self-assessed knowledge, attitudes, and educational needs regarding patients with substance use disorder. American Journal of Nursing. 2023;123(4):26-33.

o Data Analysis:
Unfortunately, the variables were treated as continuous, which is not appropriate since the CBI-6 and DDPPQ scores result from the summation of discrete Likert-type items. Furthermore, a normal distribution of data was assumed, which led to the use of parametric tests such as ANOVA and t-tests.
It is important to note that nonparametric regression models and generalized linear models exist for discrete variables. Moreover, since both CBI and DDPPQ are evaluated through Likert-type scales, it would have been ideal to compute polychoric correlations between the variables.

**Thank you for your suggestion to compute polychoric correlations for the DDPPQ and CBI-6 instruments. Although individual items on the DDPPQ and CBI-6 are Likert-type, the composite scores generated by summing all items produced continuous variables. We assessed skewness using the Shapiro–Wilk test and evaluated overall normality for both scales. The knowledge and attitudes scale demonstrated no meaningful skewness and showed a normal distribution, whereas the caring behaviors scale showed mild skewness. Given our large sample size (>30) and the absence of significant outliers in both variables, and the robustness of parametric testing to minor violations of normality assumptions, we concluded that parametric tests such as ANOVA and t-tests were appropriate for our data. We updated the data analysis section to include this note. Finally, Polychoric correlations, rather than Pearson correlations, can certainly reduce the underestimation of correlation that may occur when ordinal data are treated as continuous in Pearson’s r. However, this technique is commonly used in psychometric analysis, structural equation modeling, or factor analysis. Thank you very much for this valuable suggestion.**

**Reference:**

- Field, A. (2024). *Discovering statistics using IBM SPSS statistics*. Sage Publications.
- Lumley, T., Diehr, P., Emerson, S., & Chen, L. (2002). The importance of the normality assumption in large public health data sets. *Annual review of public health*, *23*(1), 151-169.

4. Results:
The tables show highly unbalanced group sizes for key variables. For instance: female = 114, male = 6, other = 1. This large disparity makes group comparisons statistically meaningless. The p-values in such cases have no interpretive validity.
It is recommended that group comparisons be limited to descriptive statistics only. This issue affects most variables and leads to conclusions lacking statistical weight—not only due to unequal group sizes but also because the parametric tests used do not meet the required assumptions.
As previously noted, it is suggested that the authors retain only Research Question No. 2 and compute correlations applicable to the study sample. This approach would be more consistent with a descriptive study design.

**Thank you for your valuable comment. We agree with your point about the limited statistical power to examine group differences due to small sample sizes in some groups. Since some groups remained balanced—such as age, hospital type, work experience, and past OUD experience—we thought it would be helpful to include these results in the paper to give readers additional context. We have addressed this as a major methodological limitation in the Limitations section. Regarding deleting Research question 2: Deleting this research question would significantly change the paper, and we would appreciate the editor’s advice on whether to remove this question. We are happy to make further revisions based on the editor’s suggestions.**

5. Discussion and Conclusions:
These sections should be adjusted to align with descriptive results, including correlation analyses.

**Thank you for your feedback. We have updated the discussion and conclusion sections to reflect the study results and highlighted the methodology limitations.** 
