# Supplementary material for: Association between nurses’ knowledge and attitudes and caring behaviors toward people with opioid use disorder
Source: PLoS One. 2026 Mar 12;21(3):e0325874. doi: 10.1371/journal.pone.0325874 (PMC12981468; doi:10.1371/journal.pone.0325874)
Supplement: Attachment — Submitted filename: Response to Reviewers.docx [file pone.0325874.s007.docx]

**Manuscript Number:** PONE-D-25-26604

**Title:** Association Between Nurses’ Knowledge and Attitudes and Caring Behaviors Toward People with Opioid Use Disorder

Dear Editor and Reviewers,

We thank the reviewers for their careful evaluation of our manuscript and for their constructive and thoughtful feedback. We have revised the manuscript substantially in response to these comments. Below, we provide a detailed, point-by-point response outlining how each concern was addressed. All changes are reflected in the revised manuscript.

Sincerely,

Inyene Essien-Aleksi, PhD.

**Review Comments to the Author**

**Reviewer #1**: The study “Association Between Nurses’ Knowledge and Attitudes and Caring Behaviors Toward People with Opioid Use Disorder” addresses a critical gap in hospital-based care. Its findings demonstrate that nurses’ knowledge levels and attitudes significantly influence the quality of caring behaviors toward patients with opioid use disorder (OUD). This study makes a valuable contribution to nursing science by showing how nurses’ knowledge and attitudes directly shape caring behaviors toward patients with opioid use disorder.

**We sincerely thank review #1 for the positive assessment of the manuscript.**

**Reviewer #3:** The manuscript has undergone substantial improvement and now meets the standards of clarity, coherence, and conceptual validity expected for publication. The authors acknowledge the statistical limitations transparently, and it is ultimately up to the editorial team to determine whether the current analytical approach is acceptable or whether a third review round focused exclusively on methodological refinement is warranted.

Although alternative statistical approaches—such as polychoric correlations or non-parametric models—could have enhanced the methodological rigor, I consider that: the authors’ justification for maintaining the parametric analysis is methodologically reasonable; the limitations have been appropriately integrated into the manuscript; the study’s objective is primarily exploratory and descriptive, and the findings are interpreted cautiously, without causal language.

In light of these considerations, I believe the manuscript is suitable for acceptance, provided the editorial team is comfortable with the authors’ decision to retain the current parametric analytical framework.

**We thank Reviewer #3 for their thoughtful assessment and for recognizing the improvements made to the manuscript. In response to both Reviewer #3 and Reviewer #5, we further strengthened the manuscript’s alignment with an explicitly descriptive and exploratory analytic framework. Specifically, we removed all parametric analyses and reframed the statistical approach to rely exclusively on non-parametric and ordinal-appropriate methods. We believe these revisions further reinforce the transparency and methodological coherence noted by Reviewer #3 and reduce any remaining concerns regarding analytical assumptions.**

**Reviewer #5:**  Dear Authors,
Although the manuscript has addressed some of the previously identified issues, the quantitative
analysis remains very weak. Of particular concern are the assumptions underlying the data analysis
plan.
The response variables measured by both instruments do not correspond to continuous variables.
These data are obtained through Likert-type scales and, even when multiple items of this kind are
summed, the resulting score does not constitute a numerical variable in the strict sense, nor a discrete
variable in formal statistical terms, and certainly not a continuous one.
With respect to the ANOVA analysis, this term refers to a family of experimental designs that require
the random assignment of study units to factors such as race, age group, nursing role, among others.
None of these conditions are met in the present study. Causal inference would only be feasible if the
formation of groups were truly random. Furthermore, it would be necessary to evaluate potential
interactions among factors, ensure adequate balance across groups (which is also not the case in this
manuscript), and verify that the data originate from an appropriate multivariate probability model—
or a bivariate one if only two variables are analyzed. It is essential to determine whether the data
genuinely conform to a multivariate model. Kim and Timm (2006) propose SAS routines to evaluate
multivariate normality when the data are truly continuous; however, this assumption is not met here.
Likewise, it is not methodologically appropriate to apply Pearson correlation to data that are not
continuous. Under these conditions, assessing normality is also meaningless, given that the variables
are categorical and measured on an ordinal scale. For Likert-type variables with at least five
categories, the appropriate technique for estimating associations is the polychoric correlation.
Nevertheless, reliable estimation of polychoric correlations requires large sample sizes—typically
exceeding 1,000 observations, based on simulation studies—which differs substantially from the 125
units reported in this study, a clearly insufficient number for any robust correlational analysis.
The widely circulated idea that “30 cases are sufficient” is a myth applicable only—if at all—and
very narrowly to univariate variables. The required sample size depends on the variability of the
variables and, in the case of correlations, on the magnitude of the population correlation. When the
absolute value of the correlation between two variables is close to one, very few observations are
needed; however, when correlations are close to zero, required sample sizes clearly exceed 1,000 data
points. For multivariate studies, there are no explicit references supporting the use of multiple
polychoric correlations with small sample sizes.
For all these reasons, and even though the limitations acknowledged by the authors are noted, the
most appropriate course of action would be to present this work as a descriptive, exploratory study.
It may serve as an initial step toward addressing this knowledge gap through future investigations
with larger and randomly selected samples.
Finally, it is important to emphasize that nursing is a science and, as such, must adhere strictly to the
methodological standards that guide the basic sciences. The quality of scientific evidence depends on
the rigor and robustness of the procedures used. The fact that some authors in nursing research have
employed questionable analytical strategies does not legitimize their use. It is the responsibility of the
disciplinary scientific community to safeguard the quality and rigor of published research, thereby
contributing to the strengthening and advancement of the field.
Lastly, I encourage you to review the following authors, whose work may help clarify the appropriate
statistical approach for this study:
• Kiwanuka F, Kopra J, Sak-Dankosky N, Nanyonga RC, Kvist T. Polychoric Correlation with
Ordinal Data in Nursing Research. Nursing Research. 2022;71(6):469–476.
• Kim, K., & Timm, N. (2006). Univariate and Multivariate General Linear Models: Theory
and Applications with SAS. CRC Press.

**We thank Reviewer #5 for the detailed and rigorous critique. We carefully considered all concerns raised, consulted with a senior statistician at our institution, and fully revised the data analysis plan and manuscript to address these issues. The revised manuscript now explicitly adopts an ordinal, non-parametric, and exploratory analytical framework, consistent with the reviewer’s recommendations. The key revisions are summarized below:**

1. **Removal of parametric analyses**
   All parametric tests (including ANOVA, Pearson correlation, and assumptions of normality or interval-level measurement) have been removed. The manuscript no longer treats DDPPQ or CBI-6 scores as continuous variables.
2. **Explicit treatment of DDPPQ and CBI-6 as ordinal composite measures**
   We now clearly state that both instruments are composed of Likert-type items and that summed scores were treated as **ordinal composite scores**, not continuous variables. Measures of central tendency are reported using **medians and interquartile ranges**, and no claims are made regarding interval-level measurement.
3. **Use of appropriate non-parametric methods**
   Group differences are examined using **Mann–Whitney U tests** and **Kruskal–Wallis tests**, with post hoc comparisons conducted using the **Dwass–Steel–Critchlow–Fligner method** where appropriate (via SAS). Associations between nurses’ knowledge/attitudes and caring behaviors are assessed using **Spearman’s rank correlation**, with **bootstrapped confidence intervals** to improve robustness.
4. **Adoption of median (quantile) regression**
   To examine adjusted associations, we replaced linear regression with **median (τ = 0.50) quantile regression**, which does not assume normality or interval-level data and is appropriate for skewed ordinal outcomes. This approach was selected due to the observed ceiling effects in CBI-6 scores and the absence of validated clinical cut-off points.
5. **Clear framing as descriptive and exploratory**
   Throughout the manuscript, we explicitly describe the study as **descriptive and exploratory**. All causal language has been removed, and findings are consistently interpreted as **associational** rather than inferential or causal.
6. **Transparency regarding subgroup sizes and data limitations**
   We now clearly document the merging of small subgroups, the exclusion of categories with insufficient cell sizes, and the rationale for these decisions. These limitations are discussed in detail in the Methods and Limitations sections.
7. **Expanded limitations and future directions**
   The revised Discussion and Limitations sections explicitly acknowledge the constraints of ordinal measurement, non-random sampling, subgroup imbalance, and sample size. We emphasize that the findings serve as an initial step to inform future studies with larger, randomly selected samples and more advanced modeling approaches.

We believe these revisions directly address Reviewer #5’s methodological concerns and align the manuscript with accepted statistical standards for ordinal data analysis in nursing and health sciences research. Importantly, the study’s purpose is now clearly positioned as generating preliminary, hypothesis-informing evidence rather than definitive inference. Once again, we appreciate the reviewers’ careful evaluations and believe the revised manuscript is substantially strengthened as a result of the feedback.

Thank you very much.

Best,

Inyene Essien-Aleksi
